# Supplementary material for: Natural selection favoring more transmissible HIV detected in United States molecular transmission network
Source: Nat Commun. 2019 Dec 19;10:5788. doi: 10.1038/s41467-019-13723-z (PMC6923435; doi:10.1038/s41467-019-13723-z)
Supplement: Supplementary file 1 — Supplementary Information [file 41467_2019_13723_MOESM1_ESM.docx]

**SUPPLEMENTARY INFORMATION FOR:**

**Natural Selection Favoring More Transmissible HIV Detected in United States Molecular Transmission Network**

**Authors:** Joel O. Wertheim^1,^*, Alexandra M. Oster^2^, William M. Switzer^2^, Chenhua Zhang^2,4^, Nivedha Panneer^2^, Ellsworth Campbell^2^, Neeraja Saduvala^3^, Jeffrey A. Johnson^2^, Walid Heneine^2^

**Affiliations:**

^1^Department of Medicine, University of California, San Diego

^2^Division of HIV/AIDS Prevention, Centers for Disease Control and Prevention, Atlanta

^3^ICF International, Atlanta

^4^Current Affiliation: SciMetrika LLC, Atlanta

*Corresponding author: jwertheim@ucsd.edu

**Disclaimer.** The findings and conclusions of this report are those of the authors and do not necessarily represent the official position of the Centers for Disease Control and Prevention.

**Supplementary Table 1. Relationship between individual attributes and viral load in the univariate linear regression analysis for individuals with wildtype virus in the molecular transmission network, stratified by stage of infection at diagnosis.**

| Variable | Attribute | Adjusted Beta/Significance | | | | | | | |
| --- | --- | --- | --- | --- | --- | --- | --- | --- | --- |
|  |  | Stage 0 | | Stage 1 | | Stage 2 | | Stage 3 | |
| Clustered | Yes | -0.005 |  | 0.141 | *** | -0.005 |  | 0.141 | *** |
|  | No | Ref |  | Ref |  | Ref |  | Ref |  |
| Birth sex | Male | 0.120 |  | 0.335 | *** | 0.120 |  | 0.335 | *** |
|  | Female | Ref |  | Ref |  | Ref |  | Ref |  |
| Transmission  risk factor | Male-male sexual contact | Ref |  | Ref |  | Ref |  | Ref |  |
|  | Unknown/Other | 0.145 |  | -0.195 | *** | 0.145 |  | -0.195 | *** |
|  | Heterosexual contact | -0.133 |  | -0.340 | *** | -0.133 |  | -0.340 | *** |
|  | Injection drug use | 0.333 |  | -0.104 |  | 0.333 |  | -0.104 |  |
|  | Male-male sexual contact and injection drug use | 0.213 |  | 0.087 |  | 0.213 |  | 0.087 |  |
| Race/  ethnicity | Black/African American | Ref |  | Ref |  | Ref |  | Ref |  |
|  | Hispanic/Latino | 0.118 |  | 0.153 | *** | 0.118 |  | 0.153 | *** |
|  | Other | 0.023 |  | 0.168 | *** | 0.023 |  | 0.168 | *** |
|  | White | 0.148 |  | 0.253 | *** | 0.148 |  | 0.253 | *** |
| Diagnosis  age (years) | 13-19 | -0.331 | * | -0.019 |  | -0.331 | * | -0.019 |  |
|  | 20-29 | Ref |  | Ref |  | Ref |  | Ref |  |
|  | 30-39 | 0.061 |  | 0.026 |  | 0.061 |  | 0.026 |  |
|  | 40-49 | 0.237 | * | 0.029 | *** | 0.237 | * | 0.029 | *** |
|  | 50-59 | 0.104 |  | 0.038 |  | 0.104 |  | 0.038 |  |
|  | 60+ | 0.150 |  | 0.075 | ** | 0.150 |  | 0.075 | ** |
| Δ 100 CD4^+^ count^a^ | – | -0.062 | *** | -0.002 |  | -0.062 | *** | -0.002 |  |
| Diagnosis year | – | -0.002 |  | 0.016 | *** | -0.002 |  | 0.016 | *** |

^a^Increase of 100 CD4+ cells/mm^3^

*** *p* < 0.001; ** *p* < 0.01; * *p* < 0.05

**Supplementary Table 2. Number of people from each reporting jurisdiction with an HIV *pol* sequence available for this study.**

| Jurisdiction | % of total dataset  attributable to jurisdiction^a^ |
| --- | --- |
| Alabama | 0.4 |
| Arizona | 0.5 |
| California^a^ | 1.4 |
| California–Los Angeles County | 11.0 |
| California–San Francisco | 0.5 |
| Colorado | 2.2 |
| Connecticut | 2.1 |
| Washington, D.C. | 1.7 |
| Florida | 5.7 |
| Iowa | 0.0 |
| Idaho | 0.0 |
| Illinois | 1.0 |
| Chicago | 1.1 |
| Louisiana | 2.6 |
| Maryland | 1.4 |
| Michigan | 6.8 |
| Montana | 0.0 |
| New Jersey | 0.0 |
| New York State^a^ | 2.8 |
| New York State–New York City | 29.8 |
| Oregon | 0.6 |
| Philadelphia | 1.8 |
| Puerto Rico | 0.0 |
| South Carolina | 3.7 |
| Texas | 15.3 |
| Utah | 0.1 |
| Virginia | 1.3 |
| Washington^a^ | 1.1 |
| Washington-Seattle & King County | 4.9 |
| Wisconsin | 0.2 |

^a^Includes people with non-subtype B virus and/or perinatal HIV exposure.

^b^Count does not include data submitted by other jurisdictions within the state


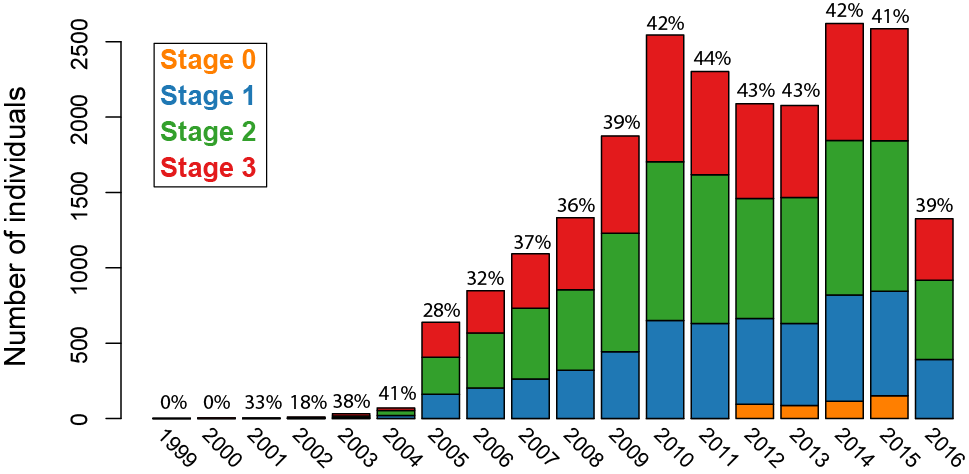


**Supplementary Figure 1. Number of individuals with HIV diagnosed per year who were ART-naïve individuals and had a wildtype partial polymerase subtype B sequence reported to the U.S. National HIV Surveillance System.** Colors indicate stage of infection at diagnosis. Percentage of individuals clustering by year denoted on top of bars. Individuals with unknown stage of infection at diagnosis not shown.

**
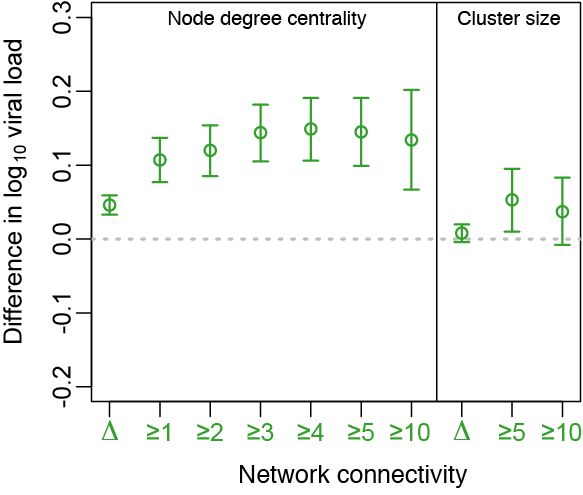
**

**Supplementary Figure 2. Difference in viral load for individuals with Stage 2 infection at diagnosis with increasing node degree centrality (i.e., number of genetically linked partners) versus non-clustered individuals and increasing cluster size versus individuals in smaller clusters.** Circles represent the mean beta for difference in log_10_ viral load in the multivariate regression analysis; error bars represent the 95% confidence intervals for these estimates. This figure presents comparable results to individuals with Stage 1 infection at diagnosis shown in Figure 2B. Node degree centrality compares individuals with at least that degree versus non-clustered individuals. Hence, node degree ≥1 in the right panel is equivalent to clustered versus non-clustered depicted in Figure 2A. Cluster size compares individuals in clusters of at least that size versus individuals in clusters of small sizes (i.e., cluster size ≥5 versus cluster size <5). Δ denotes the difference in log_10_ viral load for each increase in node degree centrality or cluster size. Network inferred at genetic distance threshold ≤0.015 substitutions/site. Sample sizes (n) for statistical tests are provided in Table 1.

**
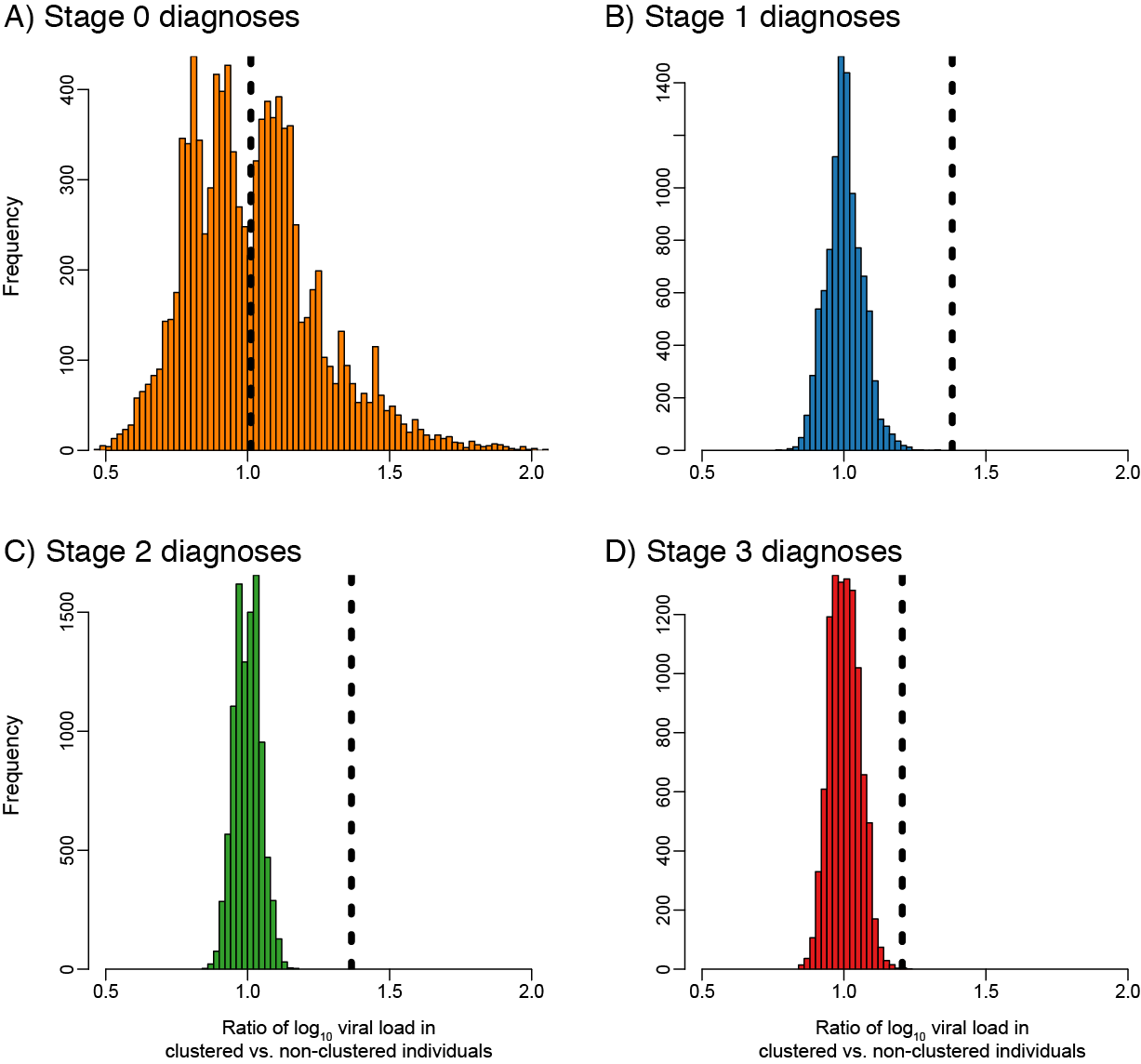
**

**Supplementary Figure 3. Permutation randomization tests across the network depicting the ratio of median viral loads in clustered and non-clustered individuals with wildtype virus.** Viral loads for individuals at each stage of infection at diagnosis are shown separately (A-D). Colored bars indicated null expectation (n=10,000 replicates) and dashed vertical black line is observed value in inferred network. Network inferred at genetic distance threshold ≤0.015 substitutions/site. Sample sizes (n) for statistical tests are provided in Table 1.


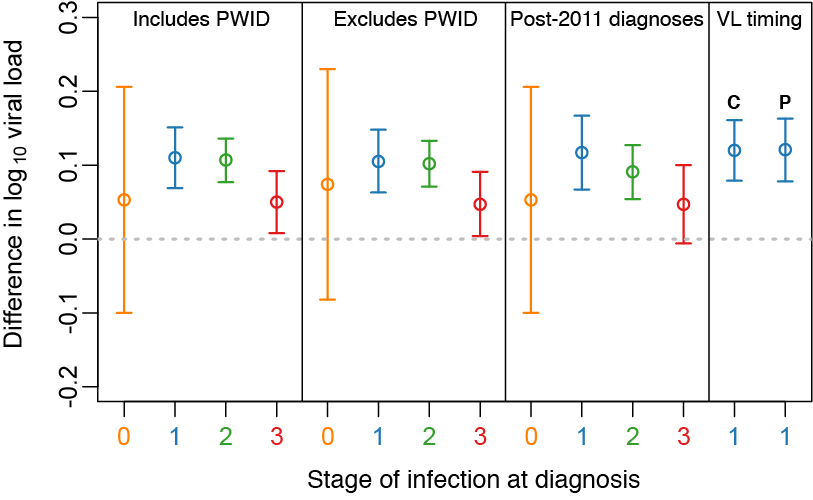


**Supplementary Figure 4. Sensitivity analysis for change in viral load (VL) for clustered versus non-clustered individuals.** Circles represent the mean beta for difference in log_10_ viral load in the multivariate regression analysis; error bars represent the 95% confidence intervals for these estimates. The first panel includes people who inject drugs (PWID) and is duplicated from Figure 2A. Sample size provided for the analysis excluding PWID: Stage 0 n = 451 people; Stage 1 n = 5512 people; Stage 2 n = 8809 people; Stage 3 n = 6767 people. Sample size provided for the analysis of post-2011 diagnoses only: Stage 0 n = 476 people; Stage 1 n = 3764 people; Stage 2 n = 5612 people; Stage 3 n = 4187 people. Alternative VL timings include (C) the VL taken closest to the reported genotype and (P) the viral load taken closest to, but not after, the reported genotype. Network inferred at genetic distance threshold ≤0.015 substitutions/site.
