# Supplementary material for: Natural selection favoring more transmissible HIV detected in United States molecular transmission network
Source: Nat Commun. 2019 Dec 19;10:5788. doi: 10.1038/s41467-019-13723-z (PMC6923435; doi:10.1038/s41467-019-13723-z)
Supplement: Supplementary file 3 — Reporting Summary [file 41467_2019_13723_MOESM3_ESM.pdf]

nature research

Corresponding author(s): Joel O. Wertheim

Last updated by author(s): Nov 21, 2019

Reporting Summary

Nature Research wishes to improve the reproducibility of the work that we publish. This form provides structure for consistency and transparency in reporting. For further information on Nature Research policies, see Authors & Referees and the Editorial Policy Checklist .

Statistics

For all statistical analyses, confirm that the following items are present in the figure legend, table legend, main text, or Methods section.

n/a	Confirmed	
☐	☒	The exact sample size (*n*) for each experimental group/condition, given as a discrete number and unit of measurement
☐	☒	A statement on whether measurements were taken from distinct samples or whether the same sample was measured repeatedly
☐	☒	The statistical test(s) used AND whether they are one- or two-sided<br>*Only common tests should be described solely by name; describe more complex techniques in the Methods section.*
☐	☒	A description of all covariates tested
☒	☐	A description of any assumptions or corrections, such as tests of normality and adjustment for multiple comparisons
☐	☒	A full description of the statistical parameters including central tendency (e.g. means) or other basic estimates (e.g. regression coefficient) AND variation (e.g. standard deviation) or associated estimates of uncertainty (e.g. confidence intervals)
☐	☒	For null hypothesis testing, the test statistic (e.g. *F*, *t*, *r*) with confidence intervals, effect sizes, degrees of freedom and *P* value noted<br>*Give P values as exact values whenever suitable.*
☒	☐	For Bayesian analysis, information on the choice of priors and Markov chain Monte Carlo settings
☒	☐	For hierarchical and complex designs, identification of the appropriate level for tests and full reporting of outcomes
☒	☐	Estimates of effect sizes (e.g. Cohen's *d*, Pearson's *r*), indicating how they were calculated

Our web collection on statistics for biologists contains articles on many of the points above.

Software and code

Policy information about availability of computer code

Data collection	These data were reported by local health departments to the Centers for Disease Control and Prevention using the Enhanced HIV/AIDS Reporting System (eHARS). These data represent the National Data Processing (NDP) as of December, 2016.
Data analysis	Data analysis was performed using HIV-TRACE (BioExt version 0.18.5; tn93 version 1.0.4) and R (version 3.4.1)

For manuscripts utilizing custom algorithms or software that are central to the research but not yet described in published literature, software must be made available to editors/reviewers. We strongly encourage code deposition in a community repository (e.g. GitHub). See the Nature Research guidelines for submitting code & software for further information.

Data

Policy information about availability of data

All manuscripts must include a data availability statement. This statement should provide the following information, where applicable:

- Accession codes, unique identifiers, or web links for publicly available datasets
- A list of figures that have associated raw data
- A description of any restrictions on data availability

The data analyzed in this article were collected and analyzed as part of CDC routine surveillance activities. Data analyzed in this study were reported by 30 state and local health departments (see Supplementary Table 2 for list). This analysis was conducted by only CDC employees and contractors. CDC is not permitted to share or distribute any surveillance data due to an assurance of confidentiality authorized under Section 308(d) of the Public Health Service Act (USA). Therefore, these data cannot be made publicly available by the authors. Each state has primary authority for determining whether their laws and regulations permit data submission to GenBank or other open databases. State and local health departments also have ability to determine whether and when these data can be shared with other researchers, as has occurred for previous studies on HIV surveillance data.

Field-specific reporting

Please select the one below that is the best fit for your research. If you are not sure, read the appropriate sections before making your selection.

☐ Life sciences ☐ Behavioural & social sciences ☒ Ecological, evolutionary & environmental sciences

For a reference copy of the document with all sections, see nature.com/documents/nr-reporting-summary-flat.pdf

Ecological, evolutionary & environmental sciences study design

All studies must disclose on these points even when the disclosure is negative.

Study description	This study was a retrospective analysis of HIV surveillance data. We used HIV sequence data to construct genetic transmission clusters and analyzed the patterns of viral load across these clusters. The unit of analysis was the individual.
Research sample	This study was conducted on HIV polymerase sequences acquired through routine drug resistance genotype screening and reported to U.S. public health departments. These data exist within the Enhanced HIV/AIDS Reporting System (eHARS). This sample is meant to be representative of the population of people living with HIV in the United States. It represents the largest repository (at the time of analysis) of HIV sequences from the United States with associated laboratory, demographic, and transmission risk data.
Sampling strategy	N/A
Data collection	Data were reported to CDC by state and local health departments
Timing and spatial scale	Data were from people with newly diagnosed HIV between 1999 through 2016. These data were reported by 30 Public Health Departments in 23 U.S. states, Washington D.C., and Puerto Rico.
Data exclusions	HIV viral sequences were excluded if they (i) had more than 5% ambiguous nucleotides or (ii) if they were sufficiently genetically similar to the HXB2 reference sequence. Viral load measurements were excluded if they were above the limit of detection/precision (1,000,000 copes/ml) or from individuals were virally suppressed at the time of HIV diagnosis (<200 copies/ml). HIV viral sequences were also excluded if they came from people who were <13 years of age at diagnosis or had reported perinatal exposure.
Reproducibility	N/A
Randomization	N/A
Blinding	N/A

Did the study involve field work? ☐ Yes ☒ No

Reporting for specific materials, systems and methods

We require information from authors about some types of materials, experimental systems and methods used in many studies. Here, indicate whether each material, system or method listed is relevant to your study. If you are not sure if a list item applies to your research, read the appropriate section before selecting a response.

Materials & experimental systems

n/a	Involved in the study	
☒	☐	Antibodies
☒	☐	Eukaryotic cell lines
☒	☐	Palaeontology
☒	☐	Animals and other organisms
☒	☐	Human research participants
☒	☐	Clinical data

Methods

n/a	Involved in the study	
☒	☐	ChIP-seq
☒	☐	Flow cytometry
☒	☐	MRI-based neuroimaging
